# Supplementary material for: Characteristics of ABCC4 and ABCG2 High Expression Subpopulations in CRC—A New Opportunity to Predict Therapy Response
Source: Cancers (Basel). 2023 Nov 28;15(23):5623. doi: 10.3390/cancers15235623 (PMC10705186; doi:10.3390/cancers15235623)
Supplement: Supplementary file 1 [file cancers-15-05623-s001.zip › cancers-2717644-supplementary.pdf]

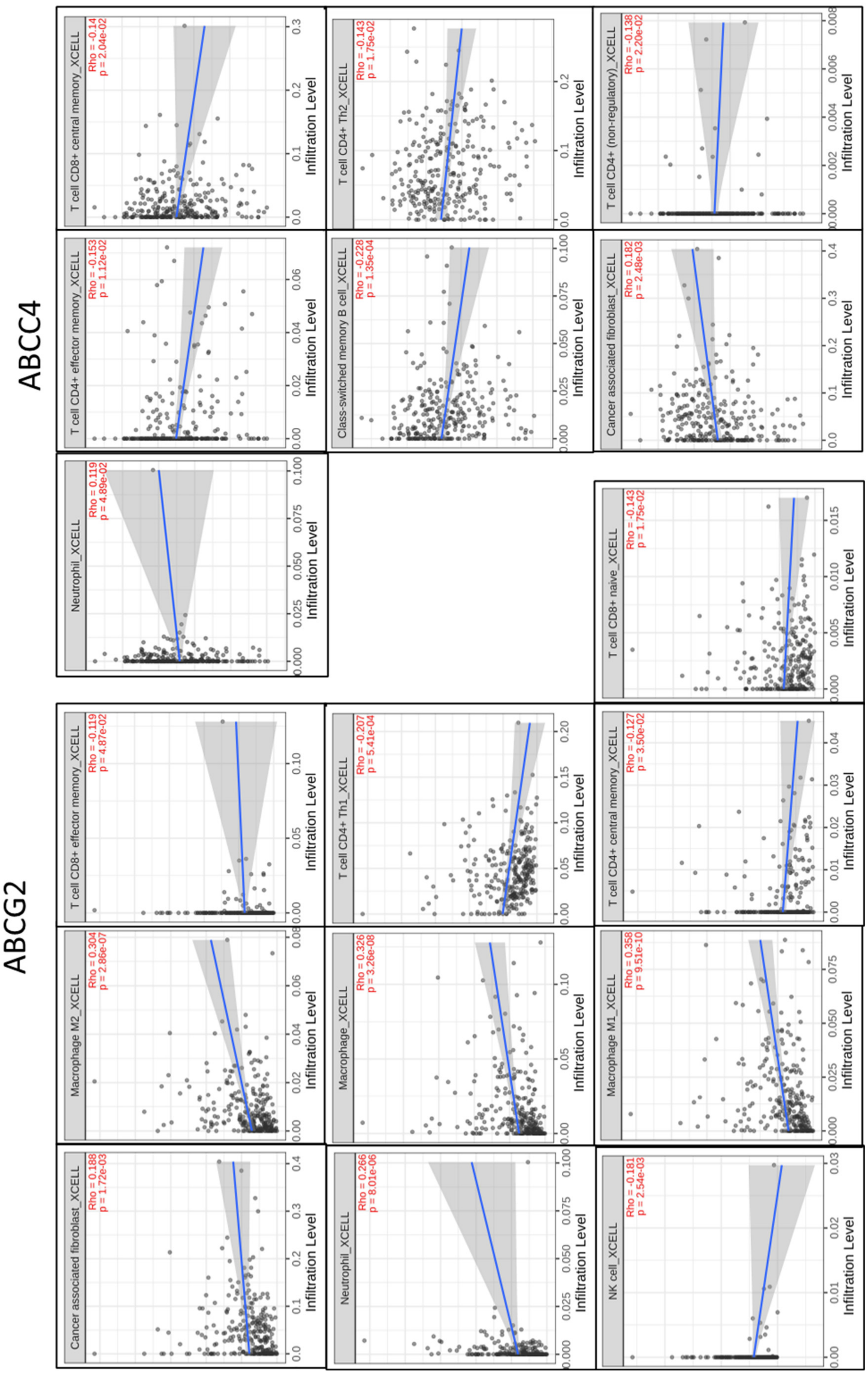

Supplementary Figure S1. Immune cell infiltration analysis was performed using the Tumor Immune Estimation Resource - TIMER2.0 (http://timer.cistrome.org/) platform and the xCell algorithm supported by the TCGA database.
